# Participation in creative workshops supports mental health consumers to share their stories of recovery: A one-year qualitative follow-up study

**DOI:** 10.1371/journal.pone.0243284

**Published:** 2020-12-03

**Authors:** Maddy Slattery, Hayley Attard, Victoria Stewart, Helena Roennfeldt, Amanda J. Wheeler

**Affiliations:** 1 School of Human Services and Social Work, Griffith University, Brisbane, Australia; 2 Faculty of Medical and Health Sciences, University of Auckland, Auckland, New Zealand; University of Sao Paulo Medical School, BRAZIL

## Abstract

Participation in creative activities has been linked with positive outcomes for people with mental illness. This longitudinal qualitative study is a one-year follow-up of eight mental health consumers who participated in a series of creative workshops in Brisbane, Australia that aimed to increase participants’ capacity and skills in sharing their stories of recovery with others. It also sought to understand successful factors of the creative workshops to inform future workshops. Semi-structured interviews gathered information regarding participants’ memories of the workshops and how they had shared their stories with others over the preceding 12 months. Interpretative phenomenological analysis identified that participants’ enjoyed being engaged in a range of creative mediums in a group setting; that peer mentor support was highly valued; and that participants’ recovery stories had become more positive and were shared more often and openly with others. Overall, participation in the creative workshops had long-lasting benefits for participants with respect to improved confidence and understanding about their illness. Future creative workshops should consider the inclusion of peer mentors with lived experience as a support for participants to reauthor their recovery story.

## Introduction

There is an increasing emphasis in mental health services on how non-traditional interventions, including art, can support service users [1, 2]. While historically art has been used in mental health assessments as a diagnostic or projective tool [3], more recently a wide range of artistic mediums (dance, music, poetry, painting, photography, and digital media) have been used in a creative workshop format to promote recovery for people experiencing mental health issues [2, 4–6]. Providing people with an opportunity to “re-author” their story through the use of creative processes and move from a primary focus on their illness to positive aspirations for their future can be an important step in the recovery process [6].

Creative workshops are delivered in both community and mental health service settings, and facilitated by health professionals, artists, or peer mentors [7, 8]. The skills of workshop facilitators and the focus of workshops may influence the experiences of participants. The active enagement of health professionals in workshops with service users can equalise power relations with service users [9]; while workshops facilitated by artists are beneficial in helping participants develop identities beyond a person with mental illness [10, 11]. The inclusion of peer mentors (someone with lived experience of mental illness) as facilitators is also important in providing participants with role models who have experienced the recovery process [12]; and encourages artistic development within an environment of mutual support [8].

A variety of benefits for attending creative workshops have been identified in a number of qualitative studies. Participation has been associated with indiviudals feeling a sense of pride and accomplishment in their artistic acivements which then can be translated into other aspects of their life [2]. For individuals who struggle with motivation, creative workshops can give them a reason to get up in the morning [13], and in doing this their confidence and self-esteem can build [14]. Creative workshops may also provide a welcome distraction from personal issues and life stressors [15]. They can also provide participants with alternative ways to explore and understand the experience of mental illness [16]. Group workshops can provide opportunities to develop social connections and a greater sense of meaning in life [17, 18]. They can foster an environment of belonging, with relationships based on mutual interests in art rather than mental illness [2, 9].

Although there are many benefits for participating in creative workshops, some challenges have been identified. Some participants have reported that while creative workshops generally elicited positive memories for them, there was the possibility of triggering negative, distressing memories as well [16]. De Vecchi et al [8] found that people who were anxious, struggled with motivation, or who had low self-esteem, felt some discomfort before attending a creative workshop and regular attendance for them was difficult. Cooper [1] has also found that some participants may feel anxious or “stuck” once workshops end if they do not have another therapy or activity to undertake.

Previous research on creative workshops has generally used a cross sectional design and included only one or two artistic mediums, typically delivered by mental health professionals and/or artists [1, 19, 20]. This paper reports a one-year follow-up of eight participants who took part in a series of creative workshops, (Your Story Matters), in Brisbane, Australia in 2015. Participants were recruited from an organisation which provides flexible funding to people with severe and persistent mental illness with complex needs. The creative workshops provided opportunities for participants to explore four artistic mediums (visual art, music, dance and creative writing) with the aim of supporting them to tell their stories of recovery and share their experience of working with services.

Eight creative workshops, each two hours in length were delivered over a three-month period. Workshops were held in a centrally located venue which had access to an outdoor space for dance and music activities. Participants explored the four artistic mediums over four separate workshops (one medium per week). These workshops were facilitated by a professional artist and supported by three peer mentors with a lived experience of mental illness. A further four workshops, attended by all the artists (one female, three male) and mentors (two females, one male), enabled participants to work one-on-one in their preferred artistic medium. Both professional artists and peer mentors had formal qualifications in human services and experience with working with people with a mental illness.

The workshops aimed to build the confidence and skills of participants to comfortably share their story of mental illness and recovery. Workshops commenced and finished with group activities, such as drumming circles that focused on connecting as a group. Each workshop included sharing of information and personal reflection by the mentors and invitations for participants to consider creative ways to express their personal stories (who we are and how we are known). Participants created individual art works, played musical instruments, wrote poems, engaged in dance movement and collectively made a mural and wrote and performed as song as a way to share their recovery stories.

Due to the potential risks identified in the literature, prior to the first workshop, artists and peer mentors attended an orientation session which focused on the structure and content of each creative workshop as well as duty of care, the expected roles and responsibilities of artists and peer mentors and information about what to do if a participant was unwell or distressed. Participants also attended an orientation session and were provided with an overview of the workshop schedule, information about the artists and peer mentors. A set of group rules was developed and agreed to by the group which aimed to promote confidentiality, inclusion and respect. Participants were also involved in a discussion of the benefits and challenges of sharing personal stories [8].

The current study had two aims: i) to explore participants’ sharing of their lived experience stories with others over the past year; and ii) explore the aspects of the creative workshops participants found most beneficial to inform similar future programs.

## Method

### Design

Individual, semi-structured interviews were used to understand participants’ memories of the creative workshops and the story-telling experiences since the completion of the workshops. An interview guide was developed by the researchers, with questions reviewed by a peer mentor. Interviews were conducted by two researchers (HA, HR). Griffith University Human Research Ethics Committee provided ethics approval (2015/370).

### Recruitment

All participants (N = 11) who attended the creative workshops in 2015 were invited to participate in the one-year follow-up; eight agreed to participate. Inclusion criteria for the original study included participants receiving integrated support services where the criteria included being aged 18 or over, experiencing severe and persistent mental illness (diagnoses such as schizophrenia, bipolar disorder) and complex needs (including issues such as homelessness, unemployment and social isolation). Participants ranged from 39–63 years (mean = 49.85, SD = 7.44); were mostly female (n = 7) and all spoke English as their first language. All participants were experiencing severe and persistent mental illness (diagnoses such as schizophrenia, bipolar disorder) and complex needs (including issues such as homelessness, unemployment and social isolation). Individual clinical diagnoses were not collected due to a concern that with such a small sample size, disclosing participants’ diagnoses may make individuals identifiable. Other studies have also shown that the results of creative workshop participation reflected similar findings across a variety of mental health diagnoses, so it was deemed unnecessary to focus on a mental health diagnosis [21].

Participants were contacted by telephone and provided with an information sheet. They were recontacted two weeks later to ascertain interest, confirm understanding of the research, obtain verbal consent, and arrange a mutually acceptable time and place (e.g. their home, café, park) for the interview. At interview completion participants were provided with a $50 gift card in appreciation of their time.

### Data collection

The interview guide (Table 1) focused on how the creative workshops had influenced participants’ decisions to share their stories and explored aspects they had found beneficial. Interviews averaged 60 minutes. Participants were given the option of having a known peer mentor attend their interview to provide support; five participants agreed. All but one interview was recorded; with one participant providing written responses.

**Table 1:** Interview topic guide.

**Sharing your story**
What are your recollections of participating in the creative workshops?
One of the goals of the workshop was to assist people to learn about sharing their stories more openly with others, can you tell me about any experience where you have shared your story since participating in the workshop?
How did the workshops prepare you for sharing your story with others?
What did you learn from the workshops that influenced your decision to share your story?
What influenced how you shared your story with others?
Since the creative workshops has how you would tell you story changed at all?
How do you feel about other people knowing you have a mental illness? Did the creative workshops influence how you feel?
Can you tell me about a time when you may have been reluctant to share your story? What were your hesitations about sharing your story?
How has sharing your story impacted on how you feel about yourself?
**Creative workshop experience**
What was your experience of the art workshop/dance workshop/music workshop/ creative writing workshop?
How did you find working with peer mentors?
What was your experience of working creatively within a group?
If you were to participate in the workshops again, what would you change to make them better?
What level of contact have you had with people from the workshops over the last 12 months?
What type of contact have you had with them? How has this contact been for you?

### Data analysis

An interpretative phenomenological analysis (IPA) approach was selected as it is ideal for exploratory studies with small sample sizes, allowing participants to tell their stories in their own words, thus making sense of their experiences [22]. In line with the IPA approach, field notes were used to document participants’ emotions and reactions. IPA aims to make sense of participants’ experiences through a process of coding, organising, integrating and interpreting data [22]. Audio recordings were transcribed verbatim and sent to participants to confirm accuracy and then entered into NVivo^©^ software [23]. Data analysis steps followed the flexible IPA guidelines which included i) multiple readings of the transcripts and making notes of researcher thoughts, observations and reflections; ii) transformation of notes into emerging themes; and iii) identification of relationships and clustering of themes [22]. Two members of the research team (HA, VS) carefully read and coded all interview transcripts and any differences and concerns regarding the coding were discussed until consensus on a set of themes was reached [24]. Overarching themes were determined by looking at what subthemes have in common and linking these together, ensuring that quotations or examples supported each theme [25].

## Results

Three main themes and eight subthemes were identified (Table 2). The themes and subthemes are discussed in the next section, supported by quotations with pseudonym identifiers.

**Table 2:** Summary of themes and subthemes.

Theme	Subthemes
**My identity, my story**	• My identity
	• Sharing my story
**Creative workshop experience**	• Artistic expression
	• Group work
	• Feeling safe and valued
	• Routine and commitment to attend
	• Connection and acceptance
	• Impact of peer mentor support
**Life since the workshops**	

### My identity, my story

This theme described the creative workshops and the impact on the way participants understood their mental illness and how they shared their recovery story with others.

#### My identity

Many participants said the creative workshops provided opportunities for them to explore their mental illness and the impact on their life, inspiring them to explore different aspects of identity and their illness, leading to a realisation that the illness was only one aspect of their identity. The creative activities provided participants with opportunities to reflect on life experiences and how these may have contributed to their illness, or exacerbated symptoms; greater acceptance then followed:

*Having a mental illness is like any other illness so it’s okay*. *That’s basically what I learnt about myself*, *that [mental illness] was okay*. *(Wendy)*

Following the workshops, some participants reported feeling less embarrassed about disclosing their mental illness, and experienced greater self-acceptance.

#### Sharing my story

Participating in the creative workshops increased comfort in sharing recovery stories. The workshops were described as safe places to explore aspects of illness and recovery experiences. Participants reported increased confidence to share their stories with family members, describing positive outcomes from sharing.

*I can talk to [family] about that now so they know I have PTSD*, *and this is the ramifications*. *I can talk to family about it more than I did before because I understand [PTSD] better now than what I did before the workshop*. *(Wendy)*

Successful sharing with family gave participants confidence to share more widely. Five participants shared their story through public presentations to students and mental health workers; many of those who spoke publicly did so on more than one occasion, suggesting that it was a positive experience. Six participants reported they would be more likely to share their story if it helped somebody else (e.g. educating others or promoting community acceptance of mental illness).

*I can be proactive in making mental health not a taboo subject*. *(Susan)*

The creative workshops also provided the opportunity to celebrate their achievements and their recovery journey.

*Being able to share your story with people*, *but not to be always negative about it*. *Just say what you have learnt and how far you’ve come*. *(Vivian)*

Although sharing stories was mostly a positive experience, all participants described barriers to sharing including the need to be selective with whom they shared.

*I’m not going to walk up to a stranger and say*, *hey I’ve got a mental illness*, *or I have PTSD… it depends on the situation and the person I’m talking to*. *(Wendy)*

Three participants expressed a reluctance to share their story because they were worried about alienating or upsetting others. Some were concerned that the information they shared would be used against them.

*I am cautious to say what I think because of the threat of being put in hospital*. *(Susan)*

Many of the participants reported that although they had not disclosed their illness prior to the workshops they now felt more confident to selectively share.

### Creative workshop experience

This theme discusses participants’ experiences of the creative workshops, and includes the subthemes of artistic expression, group work, the importance of feeling safe and supported, feeling connected and accepted by others, a commitment to attend workshops, and the impact of peer mentors.

#### Artistic expression

The range of artistic mediums (visual art, music, dance and creative writing) was valued for providing variety in ways for participants to express themselves.

*I feel [variety] was important not only for me but because people express themselves differently*. *Some people were writing*, *some people were painting*, *some people were dancing*. *I think that’s the good thing about [the workshops]*. *There was a mixture of everything*. *(Wendy)*

All but one participant discussed art as a particularly important activity. Art activities were described as a “language” for participants; a way to translate their ideas and help them to better understand and visually express feelings and memories. Dance was a new activity for many and, as such, received mixed reactions. Five participants enjoyed the opportunity to express themselves through movement and described dance activities as “*healing*”, “*calming*” and “*fun*”. However, two participants were not enamoured, with one participant describing the experience as “*terribly terrifying*”. Nonetheless, this participant made connections between her mental and physical state and identified how dance required her to “*sacrifice my rigidity*, *my protection and my safety*”, thus enabling her to connect to the impact of trauma on how she held and moved her body.

*[Dance] introduced me to a whole heap of memory that I have not processed and needs processing… insight into the enormity of how much mental health and mental illness affects my body*, *mind and soul*. *(Theresa)*

Although the dance activities induced strong emotional reactions for Theresa, she was also able to see the benefit in continuing “*it would be amazing to be able to feel like I could let go some of the control*. *It’s just we’re fighting my body*”.

All participants took part in the music activities and most reported enjoying these, but two participants noted the music was too loud.

*The drumming was too noisy for me*… *It’s not therapeutic for me*. *(Vivian)*

Of all the artistic mediums, the writing activities seemed to be the least popular with only two participants stating they really enjoyed them.

#### Group work

Most participants (n = 6) said that working as a group on artistic projects was important in having fun, sharing creative ideas, supporting each other and achieving group-based outcomes.

*It was all about us in a circle and getting that inclusion again (Graham)*.

There were some challenges to group work, however, as this was a relatively new experience for some participants. Nonetheless, the connections formed, and the support provided by group members enabled those who felt anxious to participate.

*I do suffer from social anxiety*, *so it was important because of the fact that I could actually be in a group and be able to tolerate it*. *(Vivian)*

Working in groups produced tensions amongst participants with different ideas, although most understood that this was normal in group work and they were able to tolerate differences.

#### Feeling safe and valued

Feeling part of a safe and supportive group was mentioned by all participants as an important aspect of the workshops; “*what you said in there stayed there*”. Participants reported feeling valued and welcomed when they arrived, with their individual needs acknowledged and accepted by other workshop participants. This environment led to an increased sharing of stories as participants felt accepted and not judged.

*A sense of feeling free and safe and able to express yourself without going “oh my god*, *what are they going to think*?*”*. *No judgement*. *That was what really stuck with me in my memory*. *(Vivian)*

#### Routine and commitment to attend

Four participants mentioned how the workshops helped them develop a routine. Having something to look forward to assisted participants to overcome obstacles to attending workshops: two participants travelled for two hours to attend; one was an inpatient in a mental health unit, and another was homeless.

*I had a lot of other issues at the forefront*, *but [the workshops] kept me going because I had structure in my week*. *No matter what was going on*, *even though I was homeless*, *I knew I would get up on a Tuesday and was going*. *(Wendy)*

Three participants reported missing the routine and structure when the workshops finished.

*It was strange kind of because [the workshops] were a Tuesday thing*… *so then it was like*, *oh okay*, *what am I going to do on a Tuesday now*? *(Graham)*

#### Connection and acceptance

Memories of meeting new people and social interaction were the most common responses for participants. Social connection did not always mean that participants became close friends, however, interacting with familiar people each week was valued. Four participants described feeling uncomfortable around others at first but subsequently reported feelings of friendship and support. Connections with others who had a lived experience of mental illness appeared to contribute to participants feeling accepted and equal. “*Fitting in*”, of “*being an equal*”, “*being in the same basket*”, not being a “*fish out of water*”, were sentiments that resonated strongly within the group.

*[The workshops] were the first place in a long time where I actually felt normal*. *(Wendy)*

From these social interactions, relationships were formed with four participants describing close friendships continuing after the workshops ended. These friendships proved positive for all involved, providing an opportunity to talk about mutual interests outside the shared experience of mental illness.

#### Impact of peer mentor support

The support provided by peer mentors was generally identified as positive; they shared their own journeys of recovery, highlighting commonalities and deepening connections with participants. Their stories were widely seen as inspirational with mentors considered role models.

*What [peer mentors] role modelled for me was bravery and they showed me what recovery could look like and that doors were not closing but that new doors were opening*. *(Theresa)*

Peer mentors provided different types of support. They encouraged participants to participate and consider personal preferences and meaning making through creative expressions, provided practical help with reading and writing, and emotional support.

*[Peer mentors] could pick up if you weren’t travelling well*. *A couple of days when I walked in and I was not in a good headspace*, *[peer mentors] said “how are you doing*? *Are you ok*?” *(Vivian)*

Susan reported that peer mentors gave her confidence to participate in things she thought she could otherwise not do, thus changing her response from “*what*! *I’m not doing that*” to “*yeah why not*!”

### Life since the workshops

Most participants recognised their recovery journey was ongoing. Four participants enjoyed the creative workshops so much they pursued artistic mediums after the workshops. One participant took private art lessons, another participated in community art classes and has since exhibited locally, another commenced piano lessons and one continued dance workshops. The creative workshops also inspired two participants to better understand their mental illness through completing academic study and attending community mental health events.

Four participants have engaged in public speaking events since the creative workshops including presentations for mental health staff, politicians, and university students. One participant has gained employment as a consultant at a “lived experience” organisation and regularly shares her recovery story. Keeping in contact with others from the workshops was important for most participants; however, over time the level of contact diminished.

*I posted a couple of things on there but it’s kind of stopped… I don’t know why*, *maybe people are unwell*, *maybe people have other priorities… which is kind of a shame*. *(Maria)*

## Discussion

The purpose of this study was to conduct follow-up interviews with workshop participants one year following attendance at a series of creative workshops. The interviews explored experiences of sharing recovery stories and the most beneficial aspects of the workshops. Workshop participants shared experiences of illness, loss, recovery and hopes; the workshop experience included feeling valued, connecting with others, learning about and accepting themselves, and looking toward the future.

Self-disclosure and the sharing of lived experience stories are important strategies for challenging stigma and promoting empowerment [26]. Since the creative workshops, participants reported increased confidence in sharing their recovery stories more widely. Helping others experiencing mental health difficulties or teaching others about mental illness motivated participants to share their stories. Educating others about a specific mental illness (e.g. depression, schizophrenia), creating an awareness of the prevalence of mental illness in society, or addressing myths about mental illness, were considered positive ways to use their stories in the hope of decreasing stigma. Similar to research by Van Lith, Fenner and Schofield [11] participants reported this “preparedness” to share their story was closely associated with the supportive environment created within the workshops; where they felt comfortable to share with one another.

All participants described a change in how they shared their story at the one-year follow-up. For some, their story telling increased from only sharing with mental health professionals, to sharing with their family and friends; others shared more publicly. In addition, many had altered the way they described their story; emphasising how far they had come, and what they have overcome, as opposed to focusing on the negative impact of illness. This aligns with previous research which found that sharing one’s story positively is vital to the recovery journey and to increasing an individual’s understanding of their illness [6]. Sharing the positive aspects of recovery with family was identified as important, allowing more open dialogue and a better understanding not only of the challenges, but also the strategies used to keep moving forward in their recovery.

Despite participants sharing their recovery story more widely, they described some barriers and concerns regarding how and when they shared their story. Self-disclosure was a personal decision and several factors influenced how and why stories were shared; fear about others’ reactions and discrimination were seen as risks to sharing. This is consistent with previous research which noted the difficulties in the decision to share recovery stories as disclosure can lead to stigmatisation [27]. Despite these risks, participants were able to identify positive outcomes associated with sharing their stories such as improved relationships with family, acceptance of self, and decreased shame.

The use of artistic mediums (visual art, music, dance and creative writing), provided a new language for participants to describe and understand both their illness and recovery. In line with previous research, artistic mediums were used to translate feelings and facilitate new insights [16]. Many participants described previous struggles with articulating their feelings about their illness to others, however the opportunity to explore and understand their illness through artistic mediums provided new perspectives and ways to share these new understandings with others, including family, friends and treating professionals.

Providing a range of artistic mediums was useful as some participants felt drawn to some activities rather than others. Most participants reported connecting with art activities, whilst music and dance were also popular. Finding an artistic medium, that suited individual interests, provided opportunities for different ways of expression and reflection; the range of mediums also allowed participants to try something new.

The workshops provided important opportunities for group work. Group activities allowed participants to discuss a range of interests and share creative ideas, thus increasing confidence in social situations. Research confirms that group work increases confidence as participants see themselves as worthy to take part and create change [1]. Shared experiences and the support to try new things were seen as benefits of the group activities.

Previous research has demonstrated that peer mentors support participants to engage more fully in creative workshops [8]. Through emphasising the importance of creative works and encouraging participants to try new activities, mentors fostered feelings of hope and confidence. The confidence gained by participation in the creative workshops contributed to engagement in creative activities outside of the workshop space. This is consistent with research by Makin and Gask [2] who found continued engagement in creative endeavours promotes feelings of acceptance and reduced self-stigma. While there are benefits of engaging in community-based activities, personal disclosure requires careful consideration as negative reactions from others is not uncommon [27].

Finally, the workshops provided opportunities for social interaction, resulting in strengthened social and support networks. Consistent with previous research, creating opportunities to interact with others with similar experiences results in reduced self-stigma and expanded social and support networks [28, 29]. Although friendships have been maintained for many participants, a sense of loss and disappointment was reported when this did not occur.

Our findings support providing a range of artistic mediums, allowing for participant choice. Similar to previous research by Cooper [16], one participant reported being triggered by a previous trauma experience during a dance workshop. Hence, we recommend that creative workshop participants are made aware of the potential for powerful feelings and emotions to be evoked, and that peer mentors and workshop facilitators are provided with strategies to support participants through these experiences.

### Limitations

This study was exploratory in nature and based on a small number of participants, therefore the results may not be generalisable [22]. Longitudinal studies also rely on the ability of individuals to recall their experiences of a particular event; however, these memories may change over time [30]. In the one-year follow-up interviews participants were able to discuss their experiences at length, however, some aspects were not remembered. Even though the interviews allowed for prompting and reminding of events in the creative workshops, it is possible that other experiences were forgotten. Another study limitation was that individual diagnostic information was not collected, so we cannot determine whether participants were representative of the broad range of people with mental health issues. It is also unknown if study participants were different in some way to individuals who elected not to participate in the study. Finally, although the literature has identified several negative aspects associated with workshop attendance (i.e. triggering of distressing memories [16], feelings of discomfort prior to attending workshops [9], anxiety or stuckness once workshops have ended [1]), the interview questions used in this study did not directly assess these aspects.

In conclusion, this qualitative study aimed to explore participants’ sharing of their lived experience stories with others since the completion of a series of creative workshops, and to identify which aspects were most beneficial. The study demonstrated that the creative workshops contributed to an increase, and change, in the way participants shared their stories of mental illness and recovery through improved confidence and understanding about their illness. Aspects of these creative workshops may be beneficial for the implementation of future workshops; such as the use of a variety of artistic mediums, the contribution of peer mentors and opportunities for group work.
